# Microstructure-Dependent Creep Mechanisms in Heat-Treated CZ1 Zr Alloy at 380 °C

**DOI:** 10.3390/nano15211624

**Published:** 2025-10-24

**Authors:** Haoyu Shi, Jianqiang Wang, Meiqing Chen, Pengliang Liu, Zhixuan Xia, Chenyang Lu, Rui Gao, Weiyang Li, Yujie Zhang, Zhengxiong Su, Jing Hu

**Affiliations:** 1 Department of Nuclear Science and Technology, Xi’an Jiaotong University, Xi’an 710049, China; 2 China Nuclear Power Technology Research Institute, Shenzhen 518031, Chinakylexia45@gmail.com (Z.X.);

**Keywords:** Zr alloy, creep behavior, stress exponent, microstructure, transmission electron microscopy

## Abstract

This study investigates the stress-dependent creep behavior of a CZ1 Zr alloy exhibiting two distinct microstructural states induced by different annealing treatments. Creep tests were conducted at 380 °C under applied stresses of 140 MPa and 260 MPa. CZ1-2 (fully recrystallized), characterized by coarse grains and low dislocation density, demonstrated superior creep resistance under low stress due to suppressed dislocation activity and diffusion-dominated deformation. Stress exponent analysis revealed *n* = 5 for CZ1-1 (partially recrystallized) and *n* = 10 for CZ1-2, confirming a mechanism transition from steady-state dislocation climb to power-law breakdown. TEM characterization provided direct evidence of evolving dislocation networks, stacking faults, and second-phase particle redistribution. These findings underscore the critical role of microstructural conditioning in governing creep pathways and provide a mechanistic basis for tailoring Zr alloys to stress-specific service environments in advanced nuclear applications.

## 1. Introduction

The continued pursuit of high-burnup, cost-effective, and safer nuclear energy systems has intensified the demand for improved structural materials in current and advanced light-water reactors, particularly those with enhanced resistance to irradiation and high-temperature creep. Zr alloys, widely employed as fuel cladding materials, must retain structural integrity under extreme conditions, including elevated temperatures, high irradiation doses, complex mechanical stresses, and corrosive environments [1,2]. The performance of these materials is directly linked to the safety, longevity, and economic viability of nuclear energy systems. Zr alloys are distinguished by a unique combination of properties: an exceptionally low thermal neutron absorption cross-section (0.18 × 10^−28^ m^2^), which promotes neutron economy [3]; excellent corrosion resistance due to the spontaneous formation of dense oxide films [4]; and a hexagonal close-packed crystal structure that confers mechanical stability at elevated temperatures [5]. However, over extended service lifetimes, the creep behavior of Zr alloys is highly sensitive to environmental conditions. Degradation in high-temperature creep resistance can lead to cladding deformation, and in severe cases, compromise the fission product barrier [6,7]. Accordingly, ex-reactor creep experiments have become essential for elucidating the underlying microscopic deformation mechanisms, constructing constitutive models, and predicting in-reactor performance [6,7]. Despite progress in the development of advanced Zr alloys for extreme environments, including military and aerospace applications, improving creep resistance remains a key research focus [8]. This reinforces the importance of fundamental research to guide the engineering of next-generation alloys with tailored properties.

Over the past few decades, extensive ex-reactor creep studies on Zircaloy-2 and Zircaloy-4 alloys have led to the development of constitutive models and deformation mechanism maps [9,10,11,12]. These investigations have highlighted significant stress-dependent creep behavior in Zr-Sn series alloys. At low stress levels, creep rates exhibit stress exponents of *n* ≈ 1, indicative of Coble diffusion or Harper-Dorn creep. Under higher stress, the stress exponent increases to *n* ≈ 4–5, consistent with dislocation climb mechanisms [13,14,15]. Notably, in situ transmission electron microscopy (TEM) observations by Nam et al. [15] revealed abnormal activation of prismatic slip systems at elevated temperatures, offering direct insight into Zr alloy creep mechanisms. In Nb-containing Zr alloy systems, the “viscous glide” model proposed by Murty [16] explains the strengthening effect of Nb. Dissolved Nb atoms form Cottrell atmospheres that exert drag forces on dislocations, making their motion thermally activated in intermediate stress regimes. Furthermore, recent molecular dynamics simulations [17] have shown that the formation energy of Nb-Zr vacancy complexes is ~0.35 eV lower than that of vacancies in pure Zr, providing a theoretical basis for the enhanced diffusion creep resistance conferred by Nb. Further mechanistic investigations have shown that the addition of Nb is particularly effective in improving ex-reactor creep resistance by suppressing dynamic recovery and promoting fine-scale precipitation hardening. Nb additions delay dislocation climbing through enhanced vacancy binding, while precipitated β-Nb particles contribute to Orowan-type strengthening at elevated temperatures [18]. Sn, while historically added for solid-solution strengthening, has been shown to degrade corrosion resistance in Nb-containing Zr alloys by competing with Nb for matrix solubility and accelerating second-phase coarsening during thermal aging, thus requiring careful compositional optimization [19]. Microstructural parameters such as grain size and crystallographic texture also critically affect creep behavior. Coarse-grained microstructures with basal poles tilted away from the loading direction generally exhibit improved creep resistance, while strong basal textures aligned parallel to the cladding axis can promote anisotropic deformation. As such, tailored thermomechanical treatments have been developed to refine grain orientation distributions and suppress localized strain accumulation during service [20].

Advancements in nuclear power reactor technologies, including enhanced fuel burnup, reduced fuel cycle costs, and improved safety margins, have led to stringent performance requirements for Zr alloy fuel cladding materials. Consequently, several high-performance alloys have been developed, including Zirlo, M5, E635, the HANA series, and NDA [21,22,23,24,25]. The development of advanced Zr alloys has been driven by the objective of suppressing ex-reactor creep deformation. This has been achieved through the optimization of solid solution strengthening, second-phase particle distribution, and crystallographic texture. In the domain of commercial alloys, Westinghouse’s Zirlo (Zr-1Nb-1Sn-0.1Fe) alloy has been observed to exhibit a 40% decrease in steady-state creep rate at 600 °C when compared to Zircaloy-4. This reduction is attributed to the customized Nb/Sn ratio, which has been shown to enhance solid-solution drag and refine α-phase stability [26]. AREVA’s M5 alloy (Zr-1Nb-0.12O), processed via β-quenching, exhibits enhanced creep resistance under thermal stress by stabilizing fine β-Nb precipitates and oxygen-enriched subgrain structures that hinder dislocation climb [27]. The HANA alloy series, developed by KAERI (e.g., Zr-1.5Nb-0.4Sn-0.2Fe-0.1Cr), exhibits remarkable creep-fatigue resistance between 350 and 400 °C, attributable to the refined distribution of nanoscale β-Nb particles, which function as effective barriers to dislocation motion [28]. Toshiba’s NDA alloy (Zr-0.5Nb-0.2Cu-0.2Fe) has been found to exhibit a remarkably low creep anisotropy index of 1.15 through the implementation of texture engineering techniques, such as multistep thermomechanical processing. This outcome demonstrates enhanced the uniformity of deformation under thermal loads [29].

The CZ1 alloy, a newly developed Zr alloy by China Nuclear Power Technology Research Institute (CNPRI), exhibits promising creep resistance resulting from the combined effects of alloying elements such as Cu, Sn, and Nb. However, its creep performance is strongly influenced by thermomechanical history, particularly the extent of final recrystallization. To clarify this dependence, the present study examines the creep behavior of two microstructural states—CZ1-1 (partially recrystallized) and CZ1-2 (fully recrystallized)—at 380 °C under two applied stress levels. By integrating quantitative creep data with detailed microstructural characterization of grain morphology, dislocation structures, and second-phase evolution, this work delineates how annealing-controlled microstructures dictate the active creep mechanisms under different stress regimes. The results highlight the critical role of pre-creep structural state in shaping deformation pathways and provide a mechanistic basis for optimizing annealing treatments to enhance the long-term performance of Zr cladding materials.

## 2. Experimental Methods

### 2.1. Sample Preparation

The material used in this study is a Zr alloy known as CZ1, developed by China General Nuclear Power Group (CGN, headquartered in Shenzhen, Guangdong Province, China). The nominal chemical composition of CZ1 in weight percent is 0.8–1.4 Sn, 0.1–0.3 Nb, 0.3–0.5 Fe, 0.07–0.25 Cr, and 0.05–0.3 Cu, with the remainder being Zr. To verify the alloy chemistry, area EDS analysis was performed on the matrix, with multiple measurements taken in different regions and averaged to improve accuracy. The measured values (wt.%) are: Zr 98.41, Fe 0.27, Cr 0.05, Nb 0.09, Sn 0.95, and Cu 0.22. The Sn and Cu contents fall within the nominal range for CZ1 alloy, while minor deviations are observed for Nb, Fe, and Cr. These small discrepancies are attributed to the inherent limitations of EDS quantification at low concentrations, local compositional heterogeneity (e.g., nanoscale precipitates or solute segregation), and the fact that the analysis was limited to the matrix rather than representing a bulk average. Overall, the multi-region averaged measurements confirm that the matrix composition is consistent with the nominal alloy specification, and the observed minor deviations do not affect the interpretation of the microstructural and mechanistic analysis.

The commercial CZ1 alloy tubes were manufactured through a sequence of hot extrusion, cold pilger rolling, and intermediate heat treatment at temperatures ranging from 560 to 590 °C. The final dimensions of the tubes were 9.5 mm in outer diameter and 0.57 mm in wall thickness. To introduce different microstructural states, final recrystallization annealing was performed under two distinct temperature regimes. Tubes designated as CZ1-1 were annealed at 450–480 °C, resulting in a lower degree of recrystallization, while those labeled CZ1-2 underwent annealing at 540–570 °C, resulting in a higher degree of recrystallization. All other processing parameters were kept constant. These two variants were used to investigate the influence of recrystallization degree on creep behavior.

### 2.2. Creep Testing

Internal pressure creep tests were conducted using a dedicated testing apparatus to evaluate the creep performance of the CZ1-1 and CZ1-2 tubes under different stress conditions. The tests were carried out at a temperature of 380 °C, under two applied internal pressures corresponding to stress levels of 140 MPa and 260 MPa. For comparison, pre-creep (as-received) specimens were also analyzed. The creep tests in this study were conducted at 380 °C, which corresponds to approximately 0.3 Tm of zirconium alloys (Tm ≈ 1855 °C). This homologous temperature range represents the onset of thermally activated creep processes while remaining directly relevant to the operational temperature window of zirconium alloy cladding in light-water reactors. The applied stresses were selected to reflect representative service conditions: a lower stress level of 140 MPa and a higher stress level of 260 MPa. The lower stress simulates long-term loading under typical reactor operating conditions, whereas the higher stress approximates transient or off-normal conditions where accelerated creep may occur. This combination of temperature and stress thus provides both mechanistic insight into creep deformation at a critical homologous temperature and practical relevance to the engineering performance of zirconium alloy cladding. The details of the samples and their corresponding testing conditions are summarized in Table 1.

Following the ASTM B811-13 standard [30], the hoop (circumferential) stress ($\sigma_{h}$) was calculated as $\sigma_{h}$ = p d/2 t, where p is the internal gas pressure, d is the tube’s mid-wall diameter, and t is its thickness. Creep tests were conducted under internal pressures of 17.85 MPa and 33.15 MPa, corresponding to hoop stresses of 140 MPa and 260 MPa, respectively. All tests were carried out under internal pressurization, resulting in a biaxial stress state dominated by the hoop stress. Consequently, microstructural observations from TEM foils prepared from the tube wall reflect the deformation characteristics under this hoop stress–controlled condition.

The internal pressure creep testing system primarily comprises a vacuum furnace, a pressure control module, and an external diameter measurement device. During testing, ultra-high-purity argon gas was used to generate internal pressure within the specimen, while the vacuum furnace maintained a stable thermal environment at elevated temperature. Real-time monitoring of specimen deformation was achieved using a laser micrometer with a resolution of 0.005 mm. The micrometer system includes transmitting and receiving modules configured to continuously record the change in the outer diameter during the creep process. The measured changes in diameter were then converted to hoop creep strain by calculating the relative change with respect to the initial diameter.

### 2.3. Microstructual Characterization Methods

For microstructural characterization, TEM samples were prepared using a combination of mechanical grinding and electrolytic twin-jet polishing. Initially, 3 mm × 20 mm strips were cut along the outer wall of the Zr alloy cladding tubes using an electrical discharge machining wire cutter. Subsequently, these strips were wet-ground sequentially with 100#, 400#, 800#, 1500#, 2000#, and 3000# grit silicon carbide papers to achieve a smooth surface and remove any superficial oxide layers. Due to the inherent curvature of the cut samples, careful monitoring of grinding pressure was essential to prevent the introduction of new defects or a surface stress layer that could compromise the integrity of subsequent characterization. Following initial grinding, the strips were further thinned using 5000# grit paper until a uniform thickness of approximately 30–50 μm was achieved. Disk-shaped specimens with a diameter of 3 mm were subsequently punched from these foils. Further polishing was carried out using diamond abrasive pastes with particle sizes of 6 μm, 3 μm, and 1 μm, respectively. Final thinning to electron transparency was achieved using electrolytic twin-jet polishing. This process was conducted at a temperature of −25 °C, with an applied current of 30–50 mA and a voltage of 30 V. The electrolyte used comprised a solution of 10% perchloric acid in 90% ethanol.

Following sample preparation, the alloy microstructure was characterized using a Thermo ScientificTM Talos-F200X transmission electron microscope, Waltham, MA, USA. This characterization aimed to ascertain grain morphology and size; changes in crystallographic texture under pre- and post-creep conditions; dislocation distribution and slip orientation before and after creep; morphology, size, and distribution patterns of second-phase particles.

EDS quantification was conducted using a multi-detector system with a fully rotationally symmetric design, ensuring high collection efficiency and accuracy for the sample under any tilt angle. The system is equipped with EDS mapping functionality, allowing spatially resolved compositional analysis across multiple regions of the sample. To improve reliability and representativeness, multiple measurements were taken in different regions of each phase or matrix, and the results were averaged. These conditions collectively ensure that the EDS quantification is accurate, reproducible, and representative of the sample.

## 3. Results

This section presents the experimental results and their analysis, focusing on the creep behaviors and microstructural evolution of CZ1 Zr alloys under distinct stress levels (140 MPa and 260 MPa). The findings are organized into three key areas: pre-creep microstructure, creep curve analysis, and dynamic microstructural changes during creep.

### 3.1. Pre-Creep Microstructure

**Figure 1:**
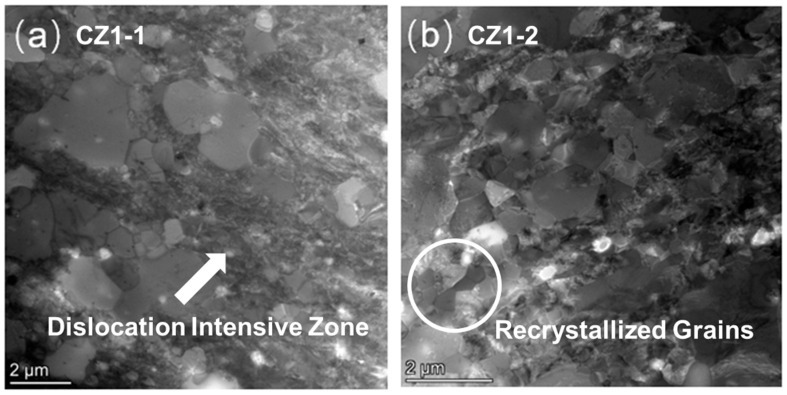
Grain morphology (STEM bright-field images) of as-received (**a**) CZ1-1 and (**b**) CZ1-2 samples.

Pre-creep microstructures of the CZ1-1 and CZ1-2 alloys were examined using STEM in bright-field mode, as shown in Figure 1. In the CZ1-1 specimen (Figure 1a), the microstructure displays a heterogeneous grain morphology characterized by irregularly shaped grains and broad regions of high dislocation density. These dislocation-rich zones appear as dark, tangled areas with streaked contrast, suggesting that a significant fraction of deformation-induced substructures remains after the final annealing treatment at 450–480 °C. The absence of widespread equiaxed grains implies that recrystallization was incomplete, and the retained dislocation configurations indicate a relatively high level of stored internal stress. Such microstructural features are expected to have a strong influence on the subsequent creep behavior, particularly in terms of dislocation accumulation and glide resistance.

In contrast, the CZ1-2 specimen (Figure 1b) reveals a relatively uniform microstructure with more well-defined, equiaxed grains and clearer grain boundaries. Several grains appear featureless in contrast and are bounded by sharper interfaces, which are typical characteristics of recrystallized grains. Compared with CZ1-1, the density of visible dislocation structures is noticeably reduced, suggesting that the higher annealing temperature (540–570 °C) promoted more complete dislocation recovery and grain boundary migration [31]. The observed differences in morphology and defect contrast provide qualitative evidence of a higher degree of recrystallization in CZ1-2. These differences are expected to affect creep deformation modes, including dislocation activity and grain-boundary-related processes, under subsequent thermal-mechanical loading.

### 3.2. Creep Behavior

**Figure 2:**
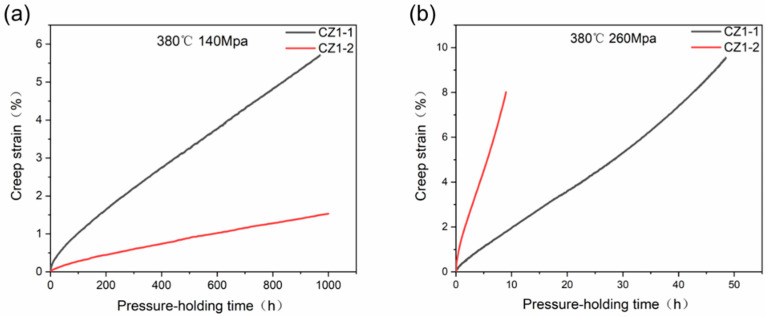
Creep curves (strain vs. time) of CZ1 Zr alloy at 380 °C: (**a**) Applied stress = 140 MPa (test duration: ~1000 h for CZ1-1, ~1000 h for CZ1-2); (**b**) Applied stress = 260 MPa (test duration: ~48.5 h for CZ1-1, ~9 h for CZ1-2). Steady-state creep windows (narrow linear segments, R^2^ > 0.995) for rate calculation are defined in Section 3.2: (**a**) CZ1-1 (200–500 h), CZ1-2 (250–550 h); (**b**) CZ1-1 (5–25 h), CZ1-2 (1–4 h). Notes: First, steady-state creep rates—with units corrected to h^−1^ (and converted to s^−1^ for reference)—are calculated from the narrow linear windows above, with an estimated uncertainty of ~±10% arising from specimen dimension measurements. Second, regarding test-to-test reproducibility: one test was conducted per condition, but reliability was ensured by vacuum furnace calibration (temperature fluctuation < ±2 °C), pressure control module calibration (deviation < ±0.1 MPa), and multi-region TEM characterization (consistent microstructural evolution. Finally, legend definitions are as follows: CZ1-1 = partially recrystallized (annealed at 450–480 °C), and CZ1-2 = fully recrystallized (annealed at 540–570 °C).

Figure 2a,b illustrate the creep curves for the CZ1-1 and CZ1-2 alloys under low (140 MPa) and high (260 MPa) stress conditions at 380 °C, respectively. Under low-stress conditions (140 MPa), CZ1-1 accumulated a total creep strain of 5.69% after 1000 h, whereas CZ1-2 exhibited a significantly lower strain of 1.56%. Steady-state creep rates were determined from narrow linear segments of the ε–t curves (small steady-state windows), selected to minimize edge fluctuations associated with transient and tertiary creep stages, with linear fits yielding R^2^ > 0.995. For CZ1-1, the steady-state window spans 200–500 h, during which strain increases from 1.85% to 4.47%, corresponding to a rate of 8.73 × 10^−5^ h^−1^ (~2.42 × 10^−8^ s^−1^). For CZ1-2, the steady-state window spans 250–550 h, with strain increasing from 0.45% to 1.13%, yielding a rate of 2.28 × 10^−5^ h^−1^ (~6.33 × 10^−9^ s^−1^). Both rates have an estimated uncertainty of ±10% due to specimen dimension measurements. These results indicate that CZ1-2, with its higher degree of recrystallization, exhibits superior resistance to low-stress creep, likely resulting from a refined microstructure and lower dislocation density. As shown in Figure 2a, the creep curve for CZ1-2 increases more gradually, indicating its enhanced resistance to deformation under these conditions. In contrast, the CZ1-1 curve displays a steeper rise in strain, reflecting its higher sensitivity to deformation at low stress levels.

Under high-stress conditions (260 MPa), the trend reverses. CZ1-2 reaches a creep strain of 8% after just 9 h, whereas CZ1-1 requires 48.5 h to reach 9.53%. Steady-state creep rates were determined from narrow linear segments of the ε–t curves (small steady-state windows, R^2^ > 0.995) to ensure precise rate evaluation. For CZ1-1, the steady-state window spans 5–25 h, during which strain increases from 2.15% to 7.63%, yielding a rate of 2.74 × 10^−3^ h^−1^ (~7.61 × 10^−7^ s^−1^). For CZ1-2, the window spans 1–4 h, with strain increasing from 1.52% to 5.39%, corresponding to a rate of 1.29 × 10^−2^ h^−1^ (~3.58 × 10^−6^ s^−1^). Both rates carry an estimated uncertainty of ±10%. These results indicate that CZ1-1 exhibits superior creep resistance under high stress, likely due to dislocation entanglement, Hall–Petch strengthening in non-recrystallized regions, and recovery-driven subgrain formation with localized low-angle boundary migration—consistent with its grain refinement trend (Table 2: average grain size reduced to 0.53 ± 0.26 μm post-260 MPa creep). As depicted in Figure 2b, the creep curve for CZ1-1 shows a faster initial increase in creep strain, indicating the onset of Stage III (accelerated creep). In contrast, CZ1-2 shows a sharp rise in strain early in the test, suggesting a rapid transition into the accelerated creep phase under high stress.

High-temperature creep in metallic materials is a thermally activated phenomenon. Assuming independent temperature and time effects on the steady-state creep rate ($\dot{\varepsilon }$), the relationship between steady-state creep rate, temperature (T), and stress (σ) generally follows the power-law equation [32]:

(1) $$\dot{\varepsilon }=A\sigma^{n}exp\left(-\frac{Q_{c}}{RT}\right)$$

where A is a material constant, $Q_{c}$ is the apparent creep activation energy, R is the gas constant, T is the absolute temperature, and *n* is the stress exponent. Taking the natural logarithm of both sides yields:

(2) $$\ln\dot{\varepsilon }=\ln A+n\ln\sigma -\frac{Q_{c}}{RT}$$

At constant temperature, let $K_{1}=\ln A-\frac{Q_{c}}{RT}$ (a constant). Equation (2) simplifies to:

(3) $$\ln\dot{\varepsilon }=K_{1}+n\ln\sigma$$

This linear relationship between lnε and lnσ at a fixed temperature allows for the determination of the stress exponent, *n*, from the slope. Higher *n* values indicate a greater stress sensitivity of the steady-state creep rate. The magnitude of n provides critical insights into the underlying creep mechanisms [33,34]: a value of approximately 1 is characteristic of diffusion-controlled mechanisms, while a value around 2 suggests grain boundary sliding. Dislocation glide is often associated with *n* ≈ 3, and dislocation climb typically falls within the *n* ≈ 4–6 range. A stress exponent exceeding 7 generally signifies power-law breakdown.

**Figure 3:**
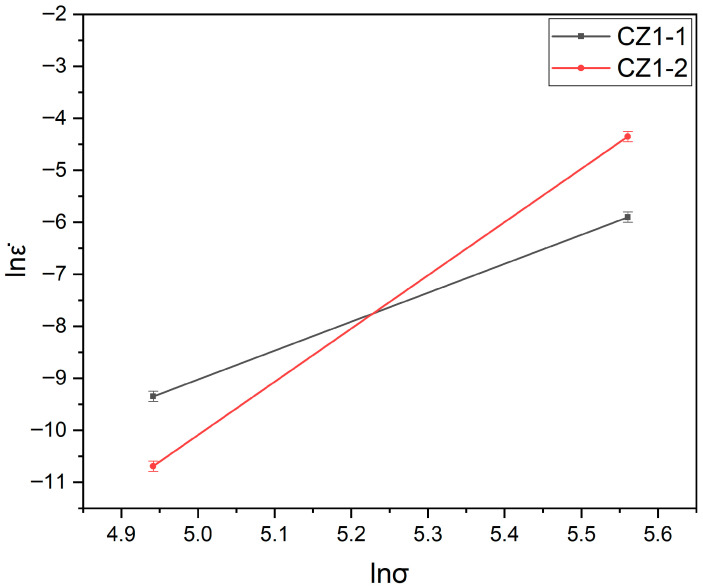
Logarithmic plot of steady-state creep rate versus stress for CZ1-1 and CZ1-2 at 380 °C.

Figure 3 shows the relationship between ln$\dot{\varepsilon }$ and ln*σ* for the CZ1-1 and CZ1-2 alloys. As shown in the figure, CZ1-1 exhibits a stress exponent of *n* ≈ 5 at 380 °C, which is consistent with a dislocation climb–dominated creep mechanism. In contrast, the fully recrystallized CZ1-2 alloy shows a distinct stress sensitivity: it maintains a power-law creep behavior (*n* ≈ 5) under 140 MPa but transitions to a much higher stress exponent (*n* ≈ 10) when the stress increases to 260 MPa. This apparent shift implies a change in the rate-controlling mechanism at high stress levels.

This interpretation is well supported by the canonical α-Zr creep mechanism map proposed by Hayes and Kassner [14], which delineates the regime boundaries for homologous temperatures (T/Tm) around 0.3—closely matching our test condition of 380 °C (T/Tm ≈ 0.29 for Zr, where Tm = 1855 °C). According to their framework, the boundary between power-law creep (dominated by dislocation climb, *n* = 4–6) and power-law breakdown (where conventional dislocation mechanisms lose rate control, *n* > 7) occurs near ~200 MPa at this temperature range. The high-stress condition applied in this study (260 MPa) lies well above this boundary, providing a mechanistically consistent explanation for the observed *n* ≈ 10.

Importantly, this mechanistic conclusion is not solely inferred from the stress exponent value. It is further corroborated by the microstructural evidence obtained for CZ1-2 under 260 MPa, including the rapid onset of tertiary creep (Figure 2b), the absence of dynamic recovery, and the formation of localized dislocation networks. These features are characteristic of the power-law breakdown regime, where steady-state dislocation climb can no longer accommodate plastic strain. The integration of stress exponent analysis, established creep mechanism frameworks, and microstructural observations thus provides a robust basis for identifying power-law breakdown at high stress.

### 3.3. Microstructural Evolution During Creep

To quantitatively compare grain size variations before and after creep, statistical measurements were conducted on both CZ1-1 and CZ1-2 alloys. For each condition, at least 200 grains were measured to determine the average grain size, as summarized in Table 2. For CZ1-1, the pre-creep sample exhibited a low degree of recrystallization, as shown in Figure 1a, with only a few grains nucleating and growing into equiaxed grains. Therefore, the average grain size was not statistically evaluated for the pre-creep condition. The results show distinct trends between low-stress (140 MPa) and high-stress (260 MPa) creep conditions. Under low stress, both alloys exhibit grain coarsening after creep exposure, whereas high-stress creep leads to grain refinement.

Grain size quantification was performed using ImageJ software (version 1.53e; National Institutes of Health, Bethesda, MD, USA), a widely used image analysis tool for microstructural statistics. For TEM bright-field images (e.g., Figure 1), thresholding and segmentation were based on grayscale contrast: grain boundaries were identified by darker grayscale values (relative to grain interiors) and manually corrected to avoid missegmentation of overlapping grains or subgrain boundaries. At least 200 grains were sampled from 3 to 5 non-overlapping TEM regions per condition to ensure statistical representativeness.

Regarding 2D-to-3D bias: Due to the use of thin TEM foils (~30–50 μm thick, Section 2.3), the measured grain sizes reflect 2D cross-sections rather than true 3D grain morphology. This may introduce minor underestimation of large, irregularly shaped grains or overestimation of small equiaxed grains—an inherent stereological limitation of thin-foil TEM analysis. To mitigate this bias, we ensured consistent foil thickness across all samples and avoided sampling regions with obvious foil bending, thereby minimizing variability in 2D cross-section representation.

This contrast is particularly evident in CZ1-1, where the average grain size decreases to 0.53 ± 0.26 μm after creep at 260 MPa (Table 2)—attributed to recovery-driven subgrain formation and localized low-angle boundary migration, which is consistent with the observed dislocation walls—in contrast to grain growth observed under 140 MPa. A similar but less pronounced trend is observed in CZ1-2. These differences reflect the influence of applied stress on substructural evolution during creep. While both alloys exhibit stress-dependent grain size changes, the magnitude of refinement and coarsening varies between the two, with CZ1-1 showing greater sensitivity to stress-induced structural modification.

**Figure 4:**
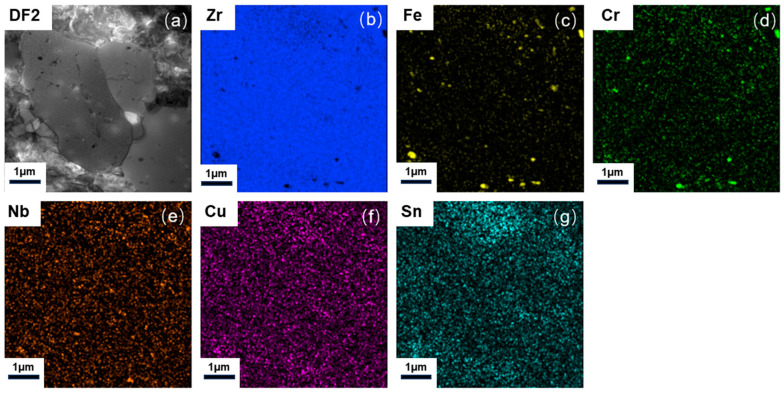
(**a**) STEM dark-field image of second-phase particles; (**b**–**g**) EDX mapping: (**b**) Zr (Kα, shown in blue), (**c**) Fe (Kα, shown in yellow), (**d**) Cr (Kα, shown in green), (**e**) Nb (Kα, shown in orange), (**f**) Cu (Kα, shown in purple), (**g**) Sn (Kα, shown in light blue) elemental distribution in pre-creep CZ1-1 alloy.

**Figure 5:**
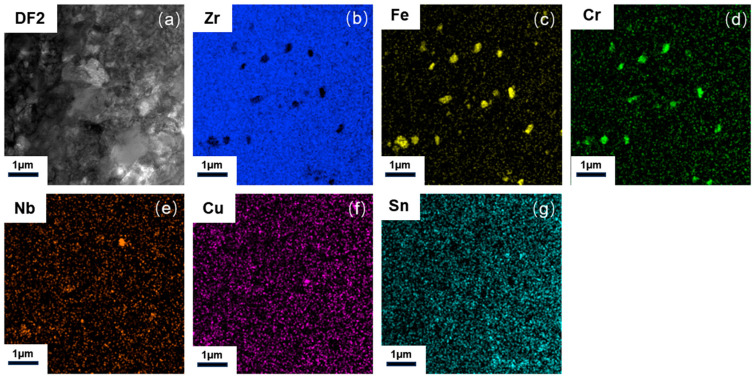
(**a**) STEM dark-field image of coarsened second-phase particles; (**b**–**g**) EDX mapping: (**b**) Zr (Kα, shown in blue), (**c**) Fe (Kα, shown in yellow), (**d**) Cr (Kα, shown in green), (**e**) Nb (Kα, shown in orange), (**f**) Cu (Kα, shown in purple), (**g**) Sn (Kα, shown in light blue) elemental distribution in CZ1-1 alloy after creep at 380 °C and 260 MPa.

**Figure 6:**
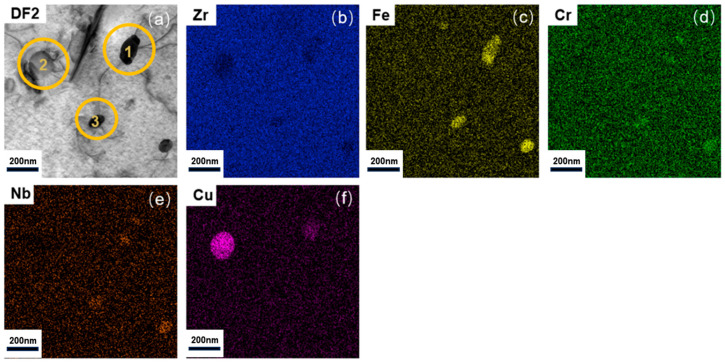
(**a**) STEM dark-field image of second-phase particles (with localized Cu enrichment); (**b**–**f**) EDX mapping: (**b**) Zr (Kα, shown in blue), (**c**) Fe (Kα, shown in yellow), (**d**) Cr (Kα, shown in green), (**e**) Nb (Kα, shown in orange), (**f**) Cu (Kα, shown in purple) elemental distribution in CZ1-2 alloy after creep at 380 °C and 140 MPa.

It is well established that fine, homogeneously distributed second-phase particles impede dislocation motion, thereby improving creep resistance [35]. However, these particles tend to coarsen under prolonged creep, diminishing their strengthening effect and increasing the creep rate. To investigate the distribution and composition of second phases in the CZ1 alloys, energy-dispersive X-ray spectroscopy mapping was performed on CZ1-1 and CZ1-2 samples under both pre- and post-creep conditions as shown in Figure 4, Figure 5 and Figure 6.

The pre-creep specimen (Figure 4) exhibits a finely dispersed distribution of second-phase particles within the Zr alloy grains. In contrast, the post-creep specimens show significant coarsening of these particles with increasing creep exposure, indicating particle growth and redistribution during deformation.

As shown in Figure 5, the EDS mapping of the CZ1-1 specimen after creep at 380 °C and 260 MPa reveals that the second-phase particles are substantially coarsened but remain primarily composed of Zr, Fe, Cr, and Nb, without any evident Cu enrichment. For comparison, Figure 6 presents the microstructure of the CZ1-2 specimen after creep at 140 MPa, where localized Cu enrichment becomes evident in certain precipitates alongside Zr-Fe-Cr-Nb phases.

The detailed EDS quantification results for representative second-phase particles (Figure 6) are summarized in Table 3. The data indicate that, after creep at 140 MPa, CZ1-2 primarily contains Zr-Fe-Cr-Nb phases (~90%) with a minor fraction of Zr-Cu phases (~10%), reflecting the stress-dependent evolution of the precipitate chemistry during creep.

Zr alloys have a hexagonal close-packed (HCP) crystal structure, where the axial ratio (c/a) is less than 1.732. This favors dislocation glide on prismatic planes. <a> dislocations typically move on prismatic planes but may cross-slip to basal or pyramidal planes at elevated temperatures or under high stress. Pile-ups of <a> dislocations can block slip on prismatic planes, leading to the activation of <c + a> dislocations, which operate on pyramidal planes and contribute to c-axis strain [36,37].

**Figure 7:**
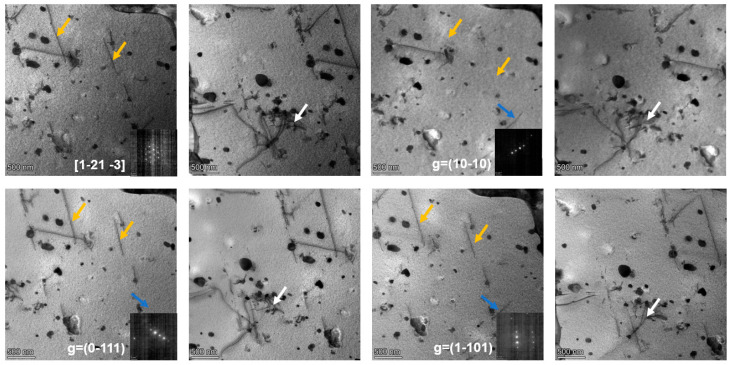
Dislocations in pre-creep CZ1-1 alloy observed via TEM bright-field (two-beam conditions) along the [1$\bar{2}$1$\bar{3}$] zone axis.

**Figure 8:**
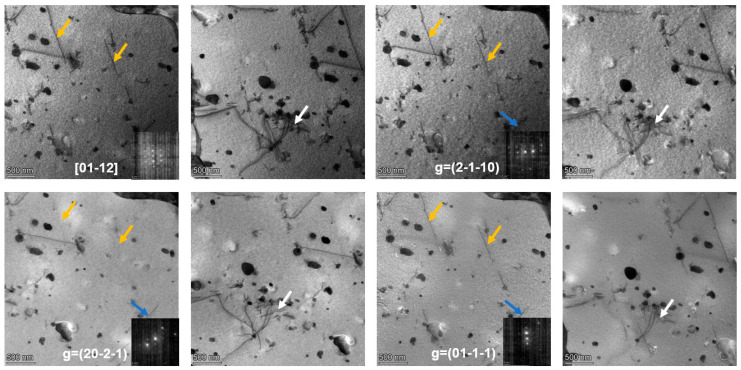
Dislocations in pre-creep CZ1-1 alloy identified via TEM bright-field (two-beam conditions) along the [01$\bar{1}$2] zone axis.

To experimentally identify the types of dislocations present in the CZ1 alloy, TEM analyses were performed. The CZ1-1 alloy before creep was selected as an example. Under the [1$\bar{2}$1$\bar{3}$] zone axis, three two-beam conditions with diffraction vectors g = (1$\bar{1}$01), g = (10$\bar{1}$0), and g = (0$\bar{1}$11) were applied, as shown in Figure 7. Three types of dislocations, marked in orange, white, and blue, were observed under these conditions. Based on the comparison between the possible dislocation types in zirconium alloys and their invisibility behavior under different g vectors, the Burgers vectors of these dislocations could not be unambiguously determined from the above observations. Therefore, additional analyses were performed under the [01$\bar{1}$2] zone axis using three distinct two-beam conditions with g = (2$\bar{1}\bar{1}$0), g = (20$\bar{2}\bar{1}$), and g = (01$\bar{1}\bar{1}$), as shown in Figure 8. According to the invisibility criteria, the blue dislocations were identified as b = <a>1/3[2$\bar{1}\bar{1}$0], the orange dislocations as b = <a>1/3[$\bar{1}$2$\bar{1}$0], and the white dislocations as b = <c + a> type.

TEM analysis was further conducted on both pre- and post-creep specimens using similar two-beam conditions to identify dislocation types via contrast extinction techniques. To ensure the clarity and consistency of dislocation characterization, Miller–Bravais notation for crystal features is uniformly defined throughout this section: crystal planes (including diffraction vectors) are denoted with parentheses (hkil) (e.g., diffraction vectors g = (1$\bar{1}$01), g = (2$\bar{1}\bar{1}$0)), and crystal directions (including Burgers vectors and zone axes) with square brackets [uvtw] (e.g., zone axis [1$\bar{2}$1$\bar{3}$], Burgers vector b = 1/3[2$\bar{1}\bar{1}$0]).

For the two-beam TEM analysis (Figure 7 and Figure 8), the invisibility criterion (g·b = 0) is explicitly correlated with observed dislocation contrast to confirm Burgers vectors. Taking pre-creep CZ1-1 as an example: under the [01$\bar{1}$2] zone axis, when the diffraction vector was set to g = (01$\bar{1}\bar{1}$) (a crystal plane), the blue-marked dislocations exhibited contrast extinction. Calculations show g·b = (0) × (2/3) + (1) × (−1/3) + (−1) × (−1/3) + (−1) × (0) = 0 (where b = 1/3[2$\bar{1}\bar{1}$0]), which satisfies the invisibility criterion. In contrast, when g = (2$\bar{1}\bar{1}$0) was applied, g·b = (2) × (2/3) + (−1) × (−1/3) + (−1) × (−1/3) + 0 × 0 = 2 ≠ 0, and the blue dislocations remained visible. This direct match between the g·b = 0 criterion and contrast changes validates the accuracy of Burgers vector identification. The summarized results of dislocation characterization under various conditions are presented in Table 4.

As can be seen from Table 4, both CZ1-1 and CZ1-2 show <a> and <c + a> dislocations before creep. Post-creep, dislocation density increases significantly, especially under high-stress creep, due to accelerated dislocation generation and pile-up in response to applied stress.

The pure <c> dislocations identified in CZ1-2 after creep at 380 °C/260 MPa (Table 4) are rarely documented in α-Zr, so their characterization was validated through multiple complementary steps using existing TEM data to ensure reliability. First, dislocations were observed and analyzed under two non-parallel zone axes—[1$\bar{2}$1$\bar{3}$] and [01$\bar{1}$2] (Figure 7 and Figure 8)—which avoided misjudgment caused by single-orientation limitations and confirmed the consistency of dislocation features across different viewing angles. Meanwhile, dual validation criteria were applied: the invisibility criterion (g·b = 0) was used to confirm the Burgers vector, and the slip plane confirmation criterion (g·(b × u) = 0, where u denotes the dislocation line direction) was employed to rule out other dislocation types. For instance, when assuming a Burgers vector of b = [0001] (pure <c>) under the [01$\bar{1}$2] zone axis, setting the diffraction vector to g = (0002) resulted in contrast extinction of the dislocations (satisfying g·b = 0); at the same time, g·(b × u) = 0 confirmed the dislocations resided on the basal plane, excluding <c + a> dislocations that require pyramidal slip planes. Additionally, the literature support further corroborates this identification: high stress (>200 MPa) has been reported to activate non-conventional slip systems in α-Zr, such as basal <c> slip [36], providing a mechanistic basis for the occurrence of pure <c> dislocations in this study. Collectively, these multi-faceted validations confirm that the identification of pure <c> dislocations in CZ1-2 under 260 MPa is not spurious, but rather a reliable reflection of the alloy’s deformation behavior under high stress.

**Figure 9:**
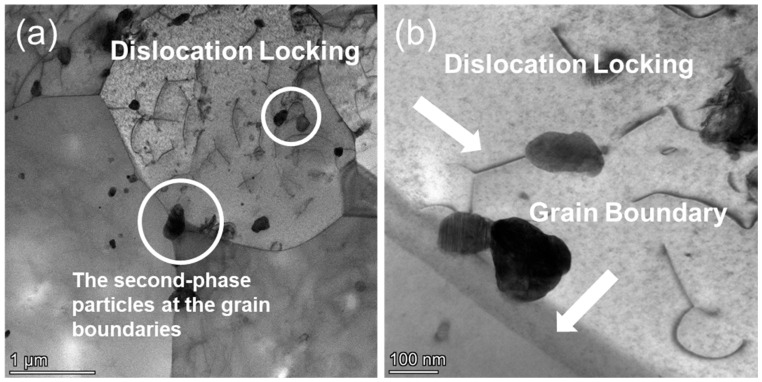
TEM bright-field images of CZ1-2 alloy after creep at 380 °C and 140 MPa: (**a**) Dislocation locking and grain-boundary second-phase particles; (**b**) Dislocation locking at grain boundaries.

In Figure 9a,b, second-phase particles are predominantly observed along grain boundaries, where their spatial distribution appears non-uniform. These particles are frequently located at triple junctions and high-angle boundaries, consistent with typical precipitation behavior at high-energy sites. Additionally, some dislocations are seen to terminate at or interact with these particles, forming localized pinning points. In certain regions, dislocation lines are arrested or deflected near particle interfaces, suggesting a structural constraint on dislocation motion. The dislocation lines are relatively sparse and appear to align along specific crystallographic directions. No significant dislocation entanglement or wall formation is observed, and the dislocation motion appears to proceed in an orderly fashion. These features indicate that under low-stress conditions, dislocation activity remains relatively unobstructed within the grain interior, with limited evidence of complex interactions or storage.

**Figure 10:**
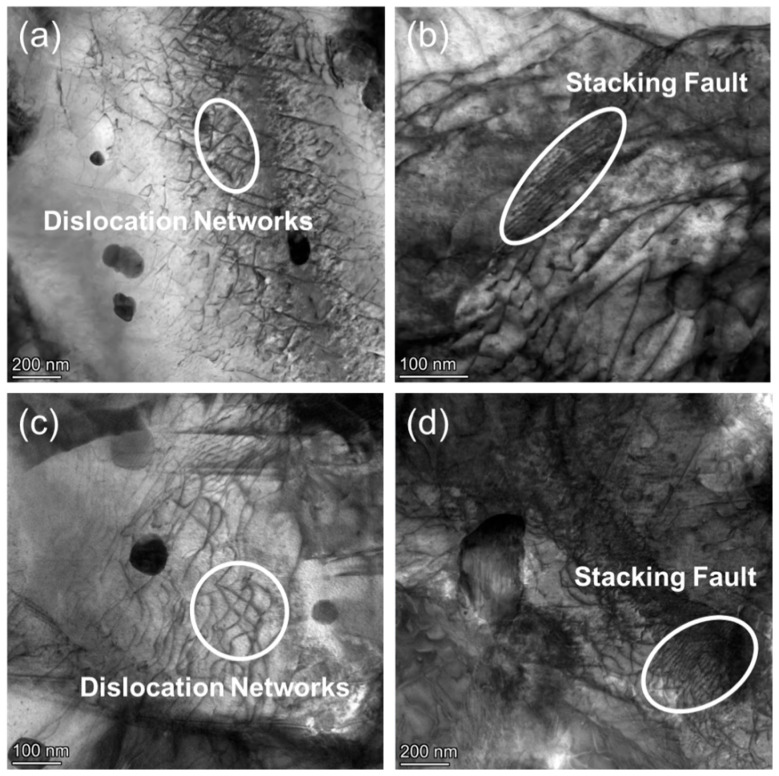
TEM bright-field images of CZ1-2 alloy after creep at 380 °C and 260 MPa: (**a**,**c**) Dislocation networks; (**b**,**d**) Stacking faults.

Figure 10a,c reveals the presence of dislocation networks within the grains of CZ1-2 alloy after creep at 260 MPa. These networks are composed of intersecting and entangled dislocations, forming locally dense structures. Compared to the low-stress condition, where dislocation lines appeared more isolated and directional, the formation of such networks suggests an increase in dislocation interactions under elevated stress. In Figure 10b,d, parallel planar features are observed within the matrix, which are identified as stacking faults. These features appear as local interruptions of the lattice contrast, extending across parts of grains or intersecting with dislocation lines. Their presence is more pronounced under higher-stress conditions and may indicate a shift in the deformation mechanism, such as increased dislocation activity or planar slip. Regarding these stacking faults (i.e., extended stacking faults, ESFs) observed in CZ1-2 (Figure 10b,d), their formation can be rationalized by the stacking fault energy (SFE) of the CZ1 alloy, supported by literature data. For Zr-Sn-Cu-Nb alloys with compositions similar to CZ1 (Section 2.1), Lin et al. [38] reported an SFE of ~15–20 mJ/m^2^—significantly lower than that of pure α-Zr (~40 mJ/m^2^). Low SFE promotes the dissociation of full dislocations into partial dislocations, which in turn generates the planar ESF contrasts captured in the TEM images. While explicit identification of fault vectors (i.e., I1 and I2, the two typical partial dislocation vectors corresponding to intrinsic and extrinsic stacking faults in hexagonal metals) was not conducted, the presence of ESFs and their correlation with low SFE align with established deformation behavior in Sn/Cu-doped Zr alloys [38], reinforcing the mechanistic interpretation of creep deformation. No evidence of twinning was observed in the present samples.

The observed microstructural features in Figure 10 differ markedly from those presented under lower stress, highlighting the evolution of dislocation structures with increased loading. The presence of both dislocation networks and stacking faults implies a shift in the deformation behavior, the nature of which will be further examined in the following chapter.

**Figure 11:**
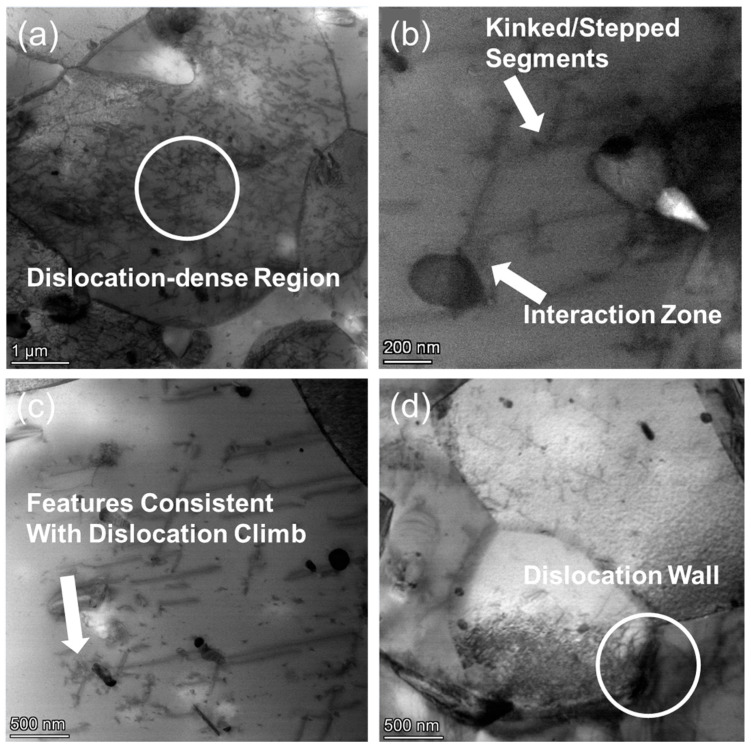
TEM images of CZ1-1 alloy after creep at 380 °C and 140 MPa: (**a**,**c**,**d**) Bright-field images (dislocation-dense regions, dislocation climb features, grain-boundary dislocation walls); (**b**) Dark-field image (kinked/stepped dislocation segments).

As shown in Figure 11b,c, the dislocation structures in the CZ1-1 alloy after creep at 140 MPa exhibit kinked and step-like segments deviating from their original glide traces. These non-planar dislocation configurations, particularly at the interaction zones with second-phase particles or local barriers, suggest that part of the dislocation motion occurred out of the primary slip plane. Similar non-planar, segmented dislocation morphologies have been interpreted as evidence of vacancy-assisted climb in high-temperature creep conditions [39]. In the present case, such features likely reflect local climb-assisted bypass of obstacles, facilitating strain accommodation and stress relaxation within dislocation-dense regions (Figure 11a).

Figure 11d shows dislocation walls near grain boundaries, where dislocations of similar character align into low-angle planar arrays. These walls interrupt otherwise homogeneous dislocation distributions and appear more frequently in CZ1-1 than in the corresponding CZ1-2 alloy under the same stress condition. The coexistence of climb-related dislocation features and organized dislocation walls indicates a distinct structural evolution pathway during low-stress creep, involving local climb-assisted rearrangement and the progressive formation of sub-grain boundaries.

**Figure 12:**
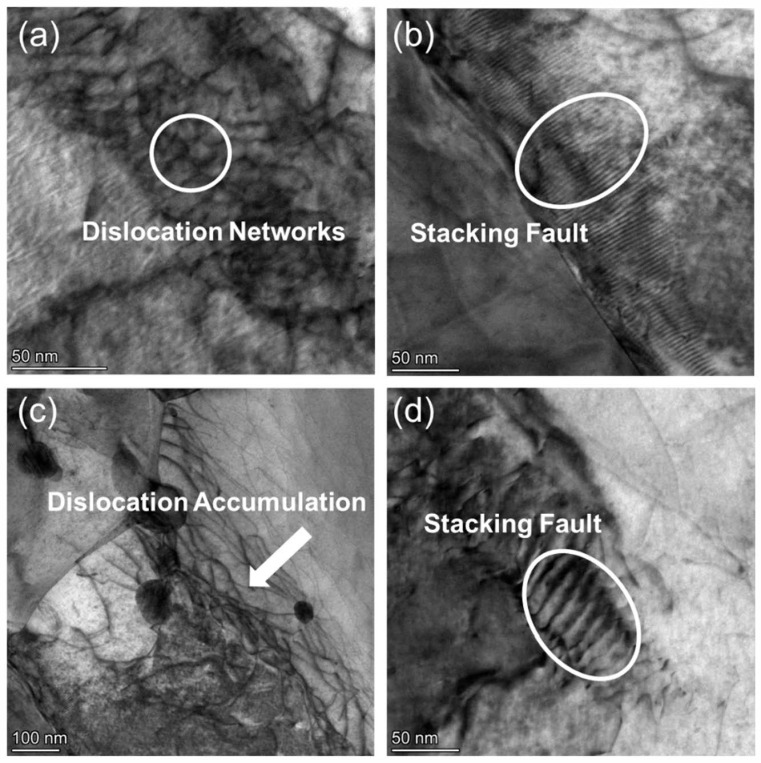
TEM bright-field images of CZ1-1 alloy after creep at 380 °C and 260 MPa: (**a**) Dislocation networks; (**b**,**d**) Stacking faults; (**c**) Dislocation accumulation.

As shown in Figure 12a, the microstructure of CZ1-1 alloy after creep at 260 MPa is characterized by the formation of dense dislocation networks and stacking faults within the grain interiors. The dislocation networks appear more entangled and spatially interconnected compared to those observed in CZ1-2 under the same conditions. These networks often span large areas and exhibit frequent intersections and junctions, reflecting a higher degree of dislocation accumulation (Figure 12c).

Stacking faults are also visible in Figure 12b,d as narrow, planar contrast features extending across grains. These defects are more pronounced and frequent in CZ1-1 than in CZ1-2, where their appearance is comparatively sparse. The combined presence of intricate dislocation networks and well-defined stacking faults distinguishes CZ1-1 from CZ1-2 in terms of microstructural response to high-stress deformation. While both alloys develop similar types of defects under elevated stress, the internal dislocation configuration in CZ1-1 reveals a more complex spatial arrangement and higher structural heterogeneity.

## 4. Discussion

### 4.1. Stress-Dependent Creep Mechanism Transition Governed by Microstructure and Rate Sensitivity

The contrasting creep behavior of CZ1-1 and CZ1-2 arises from their distinct initial microstructures, shaped by the final annealing process. The CZ1-1 alloy, annealed at lower temperatures, retains a partially recrystallized structure with high dislocation density and internal strain energy, whereas CZ1-2 undergoes full recrystallization and exhibits a uniform, equiaxed grain morphology with significantly reduced defect density. These differences affect not only the material’s initial creep resistance but also its ability to accommodate long-term deformation through internal structural evolution.

Quantitative analysis further supports this divergence. The stress exponent at 380 °C was determined to be 5 for CZ1-1 and 10 for CZ1-2, based on the linear fitting of $\ln\dot{\varepsilon }$ vs. ln*σ*. The value of *n* = 5 in CZ1-1 is consistent with a climb-assisted dislocation creep mechanism, as supported by the observed non-planar dislocation configurations, subgrain formation, and entangled networks. In contrast, the unusually high *n* = 10 for CZ1-2 suggests power-law breakdown, a regime where conventional dislocation-based deformation mechanisms lose rate-controlling influence. This anomalous rate sensitivity reflects the inability of CZ1-2’s stable structure to adapt under high stress, leading to abrupt strain localization, rapid tertiary creep onset, and a breakdown of steady-state behavior.

These findings are consistent with previous studies on commercial zirconium alloys. For instance, Zircaloy-2 exhibits a stress exponent of approximately 4.8, indicating a dislocation climb-dominated creep mechanism. Similarly, Zircaloy-4 has a stress exponent of around 5.0, also suggesting a dislocation climb-dominated creep mechanism [14]. In contrast, the unusually high stress exponent observed in CZ1-2 highlights the importance of microstructural adaptability in governing creep behavior.

Thus, the creep mechanism is highly sensitive not only to applied stress but also to the underlying microstructure’s capacity for dynamic response. CZ1-1, despite higher initial defect content, demonstrates greater resilience at elevated stress due to its ability to undergo structural reconfiguration during deformation. These findings emphasize that the evaluation of steady-state creep curves alone is insufficient to explain long-term performance; coupling rate sensitivity analysis with microstructural evolution is essential to accurately reveal dominant deformation mechanisms.

### 4.2. Interactions Between Dislocation Kinetics, Dynamic Recovery, and Precipitate Evolution

TEM observations indicate that under 260 MPa, CZ1-1 forms dense dislocation networks and undergoes recovery-driven subgrain formation with localized low-angle boundary migration—consistent with the dislocation walls (Figure 11d) and grain refinement in Table 2 (0.53 ± 0.26 μm post-creep). This substructural rearrangement increases grain boundary area and hinders dislocation motion, collectively enhancing its creep resistance. The evolution of these structures points to an active recovery–recrystallization mechanism, where dislocation climb facilitates <c + a> slip and supports axial strain accommodation in the HCP lattice. The synergy between glide and climb mechanisms underlies the extended secondary creep regime observed in CZ1-1. In contrast, CZ1-2 lacks such adaptive capacity; although its initial structure is well-ordered, it does not exhibit dynamic recovery or boundary migration during high-stress exposure. As a result, deformation proceeds via rapid, unimpeded dislocation glide with limited structural resistance, accelerating creep damage.

This mechanism is further modulated by second-phase particles. In both alloys, Zr-(Fe,Cr) and Zr–Cu precipitates contribute to grain boundary pinning, especially under low stress [38,40]. However, EDS analysis reveals that CZ1-2 accumulates Cu-rich particles after creep, likely due to stress-assisted solute segregation. These particles may initially provide a solute-drag effect, suppressing dislocation motion. Yet, this precipitation hardening is inherently time-dependent: prolonged exposure promotes particle coarsening, which diminishes their pinning effectiveness. The absence of new defect structures in CZ1-2 means there is no compensatory strengthening as particle-based barriers weaken, rendering the alloy more vulnerable in long-term creep.

Moreover, the presence of extended stacking faults in CZ1-2 across both stress levels implies a relatively low SFE, likely influenced by Sn and Cu content. Reduced SFE encourages dislocation dissociation and suppresses cross-slip, favoring diffusion-mediated climb rather than sustained glide. While beneficial under low-stress, quasi-static conditions, this also limits the activation of complex slip systems needed for creep accommodation at higher stress. In contrast, CZ1-1 displays more diversified slip and recovery structures, highlighting the importance of microstructural heterogeneity in sustaining long-term deformation resistance.

### 4.3. Mechanism-Guided Microstructural Design Strategy for Creep Optimization

The integration of stress exponent analysis, TEM-based microstructural evidence, and second-phase behavior leads to a unified framework linking microstructure to creep mechanism across stress regimes. Under low stress (140 MPa), the recrystallized CZ1-2 alloy performs well due to its coarse, stable grains, low dislocation density, and finely dispersed precipitates, all of which favor diffusion-controlled mechanisms such as Harper–Dorn creep. Its high dimensional stability during early creep stages underscores the advantage of structural cleanliness under moderate loads.

However, this advantage diminishes at elevated stress levels (260 MPa). The same microstructure that resists early deformation becomes structurally inert, unable to accommodate increased strain through dynamic recovery or substructure formation. Consequently, the creep rate accelerates rapidly, and damage accumulates without internal mitigation. By contrast, CZ1-1, with its inherited defect network and dynamic substructural adaptation capacity (recovery-driven subgrain formation), sustains dislocation rearrangement, boundary migration, and strain partitioning—traits essential for resisting high-stress creep. This dynamic adaptability enables the alloy to maintain moderate creep rates despite its initially less “perfect” structure.

These findings suggest that creep resistance in Zr alloys cannot be achieved through microstructural optimization for a single stress regime. Instead, the alloy’s microstructure must be matched to its operational context. For long-term service under low to moderate loads, a fully recrystallized, stable grain structure offers ideal stability. For conditions involving thermal gradients, transient stresses, or elevated loads, partial recrystallization with recoverable dislocation networks and latent structural mobility is essential. This dual-mode design principle, balancing initial strength with long-term evolvability, provides a practical roadmap for tailoring high-performance Zr cladding alloys in advanced nuclear systems.

## 5. Conclusions

This study demonstrates that the creep behavior of the CZ1 zirconium alloy at 380 °C is strongly governed by annealing-controlled microstructural states and applied stress. At low stress (140 MPa), the fully recrystallized CZ1-2 exhibits superior creep resistance, attributed to coarse stable grains, reduced dislocation density, and effective second-phase pinning. Under high stress (260 MPa), the partially recrystallized CZ1-1 outperforms CZ1-2, showing a stress exponent of *n* = 5 and microstructural features indicative of climb-assisted dislocation motion, recovery-driven subgrain formation with localized low-angle boundary migration, and in situ grain refinement (Table 2)—all of which enhance long-term creep resistance.

These results reveal a stress-sensitive mechanism transition in the CZ1 alloy: fully recrystallized states are optimal for long-term, low-stress service, whereas partially recrystallized structures are advantageous under high-stress or transient conditions due to their dynamic adaptability. The findings provide a mechanistic framework for tailoring zirconium alloy cladding microstructures to anticipated service regimes and contribute to the broader understanding of creep behavior in HCP alloys.

Future work should address intermediate stress regimes and irradiation effects, integrating in situ characterization and advanced modeling to extend these insights toward the design of next-generation Zr alloys for nuclear applications.

## Figures and Tables

**Table 1:** Experimental conditions for creep testing.

Sample Number	Sample Name	Creep Test Condition
1	CZ1-1	380 °C, 140 MPa
2	CZ1-1	380 °C, 260 MPa
3	CZ1-2	380 °C, 140 MPa
4	CZ1-2	380 °C, 260 MPa

**Table 2:** Average grain size (μm) of Zr alloys before and after creep at 380 °C.

Sample	Pre-Creep	380 °C/140 MPa	380 °C/260 MPa
CZ1-1	/	2.98 ± 1.38	0.53 ± 0.26
CZ1-2	0.76 ± 0.62	2.72 ± 0.92	1.19 ± 0.55

**Table 3:** Elemental composition of second-phase particles in CZ1-2 after 380 °C/140 MPa creep.

Region		Zr	Fe	Cr	Nb	Cu
Matrix	at.%	98.61	0.12	0.15	0.27	0.86
	wt.%	98.97	0.07	0.09	0.28	0.60
Area 1	at.%	82.67	9.35	0.76	1.43	5.79
	wt.%	87.65	6.07	0.46	1.55	4.28
Area 2	at.%	77.63	1.63	0.28	0.86	19.61
	wt.%	83.19	1.07	0.17	0.94	14.64
Area 3	at.%	76.41	11.55	3.37	6.95	1.72
	wt.%	81.57	7.55	2.05	7.56	1.28

**Table 4:** Dislocation types in CZ1-1 and CZ1-2 under pre- and post-creep conditions.

Sample	Creep Condition	Dislocation Types
CZ1-1	Pre-creep	<a>1/3[2$\bar{1}\bar{1}$0], <a>1/3[$\bar{1}$2$\bar{1}$0], <c + a>
	380 °C/260 MPa	<a>1/3[2$\bar{1}\bar{1}$0] <c + a>
CZ1-2	Pre-creep	<a>1/3[2$\bar{1}\bar{1}$0], <a>1/3[$\bar{1}$2$\bar{1}$0], <c + a>[$\bar{1}\bar{1}$23]
	380 °C/140 MPa	<a>1/3[2$\bar{1}\bar{1}$0], <a>1/3[$\bar{1}\bar{1}$20]
	380 °C/260 MPa	<a>1/3[2$\bar{1}\bar{1}$0], <a>1/3[$\bar{1}$2$\bar{1}$0], <c + a>, <c>

## Data Availability

The original contributions presented in this study are included in the article material. Further inquiries can be directed to the corresponding author(s).
